# Multi-Objective Optimal Planning for Distribution Network Considering the Uncertainty of PV Power and Line-Switch State

**DOI:** 10.3390/s22134927

**Published:** 2022-06-29

**Authors:** Li Gong, Xianpei Wang, Meng Tian, Hongtai Yao, Jiachuan Long

**Affiliations:** 1 Electronic Information School, Wuhan University, Wuhan 430072, China; ligong@whu.edu.cn (L.G.); mengtian@whu.edu.cn (M.T.); hongtaiyao@whu.edu.cn (H.Y.); 2 School of Electronics and Information Engineering, Wuhan Donghu University, Wuhan 430212, China; longjc@whu.edu.cn

**Keywords:** distribution network, PV power, LHS, distribution network reconfiguration, line-switch state

## Abstract

With the construction of the smart grid, the distribution network with high penetration of the photovoltaic (PV) generator relies more and more on cyber systems to achieve active control; thus, the uncertainty of PV power and the line-switch state will inevitably affect the distribution network. To avoid the situation, a min–max multi-objective two-level planning model is proposed. Firstly, the uncertainty of PV power is considered, and a multi-time PV power model is established. Followed by the analysis of the line-switch state uncertainty in the distribution network, and according to Claude Shannon’s information theory, the line-switch state uncertainty model is established under multiple scenarios. After the distribution network reconfiguration, the Latin hypercube sampling (LHS) method is used to determine the line-switch state when the uncertainty budget is different. Finally, considering the worstcase by the uncertainty of PV power and line-switch status, the control model is proposed to improve the stability of the distribution network with the minimal maintenance cost. The model feasibility is verified by the test system and the characteristics of PV power uncertainty, the line-switch state uncertainty is analyzed, and the influence of the scheduling strategy is discussed, thus providing practical technical support for the distribution network.

## 1. Introduction

With the application of various intelligent technologies, the smart grid has gradually developed into a cyber-physical system. As an important part of the smart grid, the distribution network can provide basic support to ensure the quality of power supply and improve the electrification level, and its intelligent research has made great progress [1]. However, due to the increasing access of distributed generators (DGs), intelligent sensors, and communication devices, the distribution network is facing huge challenges. Particularly, the uncertainty of photovoltaic (PV) power will lead to frequent changes in load voltage, and the distribution network thus will rely more on the operation of line switches. If the status of the line switch cannot be determined, there will be a series of power safety problems in serious cases [2,3], such as the cause of the load losses, and even the radial topology of the distribution network will change. On 13 May 2021, Taiwan Province, China experienced a power outage caused by the uncertainty of PV power, affecting 13.19 million residents. At the beginning of the power outage, about 2 million residents had restricted access to electricity. During the outage, the line switches failed to operate properly, then dispatchers were unable to determine the actual line status, further leading to the expansion of the outage. Ultimately, the load losses caused by the accident amounted to 3.5 million KW.

To cope with the above problems, the distribution network has received widespread attention, which can provide active control and management of DG (e.g., PV) to improve power quality. Thus, many scholars have focused on the development of the distribution network: Jiao et al. proposed a distributed coordinated voltage control scheme for distribution networks with DG and the on-load tap changer; through the coordination of DGs and other devices, the distributed coordinated voltage control is achieved and the computation burden is mitigated [4]. Wang et al. present a uniform control strategy for the bidirectional ac/dc interlinking converter hierarchical controlled hybrid microgrid; the negative consequences can be avoided, and the strategy could be applicable for the hierarchical controlled hybrid microgrid [5]. Sedghi et al. aim at improving the capability of DG planning, then an improved PSO algorithm is proposed to minimize the investment cost and operating cost; meanwhile, the constraints of technology, variable load, DG, and energy storage are considered [6]. Cupelli et al. propose a data-driven adaptive control for power electronic interfaced distributed energy resources to guarantee a good performance and bus voltage stability without prior knowledge of the uncertainties, while Aziz et al. have achieved the control of the voltage across the low-voltage network, making the low-voltage system more PV friendly [7,8]. For the security of distribution network, Shafiee et al. propose a comprehensive model to study the plug-in hybrid electric vehicle impact on distribution systems; the sensitivity analysis is performed to demonstrate the effects of operation modes on the network load profile [9]. Kharrazi et al. investigate the application of the supervisory control of the discrete event system to the management and control of a custom power park, and the developed systematic method can be applied to several control problems in microgrids [10]. Additionally, Liu et al. point out that the security of the distribution network is vulnerable to the cyber system; then, an analytical method is presented to quantify the impact of cyber faults considering the functionality validity during distribution automation [11]. Garmrudi et al. believe the islanding is one of the problems that arise with integrating these resources into the distribution system; if no effort is made, it will cause great damage to the distribution network [12].

The above literature focuses on control methods, DG planning, demand management, and the security of the distribution network; however, the impact of PV power uncertainty and line-switch state uncertainty are less concerned. Currently, the impact of PV power uncertainty is mainly analyzed from the perspective of security risk assessment, while line-switch state uncertainty is analyzed from the perspective of system reliability assessment. Aghamohamadi et al. present a two-stage adaptive robust optimization while considering the uncertainty of the PV power, and the immunization of the model against uncertainties is justified by testing the obtained solutions against 36,500 trial uncertainty scenarios in a postevent analysis [13]. Scolari et al. propose and validate a comprehensive method to assess the overall PV power uncertainties, and the uncertainties are quantified [14]. Wen et al. present two probabilistic approaches based on the bootstrap method and the quantile regression method, to estimate the uncertainty associated with solar PV power point forecasts [15]. From an assessment view, the impact of the cyber system (such as communication infrastructures and operation infrastructures) on the distribution network are discussed in detail [16,17,18]. Cintuglu et al. show that the line switch is an important component of the communication system infrastructures [19]. Liu et al. propose a multi-objective optimization approach to address the multiple solutions of line switching and verify the effectiveness of the proposed approach [20].

Further, researchers consider the impact of PV power uncertainty and switch-state uncertainty from the perspective of distribution network reconfiguration. Zheng et al. propose the distributionally robust model to obtain the optimal configuration under the worstcase and point out that uncertainties of DG power should be considered before determining the status of switches [21]. Based on the active cyber-physical distribution network, Liu et al. establish the cyber subsystem model to describe the performance in distribution communication; line switches are used for quantifying the interactions between subsystems [22]. This shows that the existing methods achieve good results; the mathematical optimization and algorithmic prediction are used to reduce the impact caused by PV power uncertainty; and through modeling of information disturbances and faults in the cyber system, the specific form of switch state uncertainty is characterized. Based on them, the impact caused by different scenarios and factors is analyzed. However, these ideas cannot accurately analyze the distribution network characteristics when the uncertainty of PV power and line-switch state act together and cannot provide a reference basis for schedulers.

To solve the above problems, we propose a min–max multi-objective two-level planning model to improve the security of the distribution network. Its objective is to reduce the impact caused by the uncertainty of PV power and the line-switch state. First, we propose a multi-time PV power model; its objective is to maximize PV power cost, and we use different time periods of PV power errors to characterize the uncertainty of PV power. Then, according to the form of line-switch state uncertainty, we propose the line-switch state uncertainty model based on Claude Shannon’s information theory, and we use the Latin hypercube sampling (LHS) method to maximize failure cost after distribution network reconfiguration; the worst-case scenario is simulated. Finally, we propose the control model with the objective of the minimum maintenance cost and verify the effectiveness of the proposed model.

The rest of the paper is organized as follows. Section 2 displays the model of PV power uncertainty. Section 3 displays the model of line-switch state uncertainty. Section 4 displays the min–max multi-objective two-level planning model. Numerical results are presented in Section 5. Section 6 concludes the paper.

## 2. Model of PV Power Uncertainty

As a disturbance source in the distribution network, the uncertainty of PV power is difficult to predict, so there is an urgent need to establish the PV power uncertainty model.

### 2.1. The Model of PV Power

**Figure 1:**
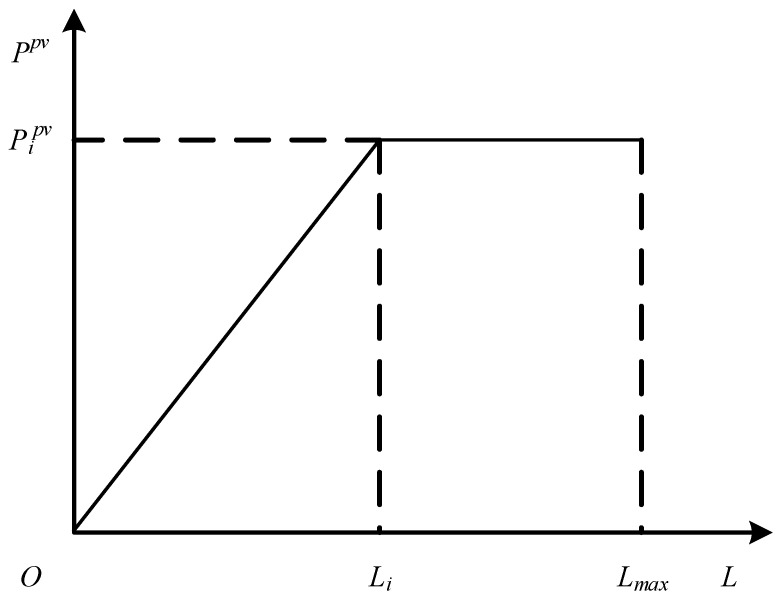
The characteristics of PV power.

PV generally includes the solar panel, the solar controller, the battery, and solar inverter. The solar panel is the core part, whose function is to convert the sun’s radiation into electrical energy, so the output of PV power is closely related to the solar irradiance. The relationship is shown in Figure 1, and its functional expression is referred to in Equation (1) [23].

(1) $$P^{pv}=\left\{\begin{array}{c} P_{rt}^{pv}*L/L_{rt}\;0\leq L\leq L_{rt}\\ P_{rt}^{pv}\;L_{rt}\leq L\leq L_{\max} \end{array}\right.$$

where $P^{pv}$ is the PV power, $P_{rt}^{pv}$ is the rated PV power, *L* is the solar irradiance, $L_{rt}$ is the rated solar irradiance, and $L_{\max}$ is the maximum solar irradiance.

**Figure 2:**
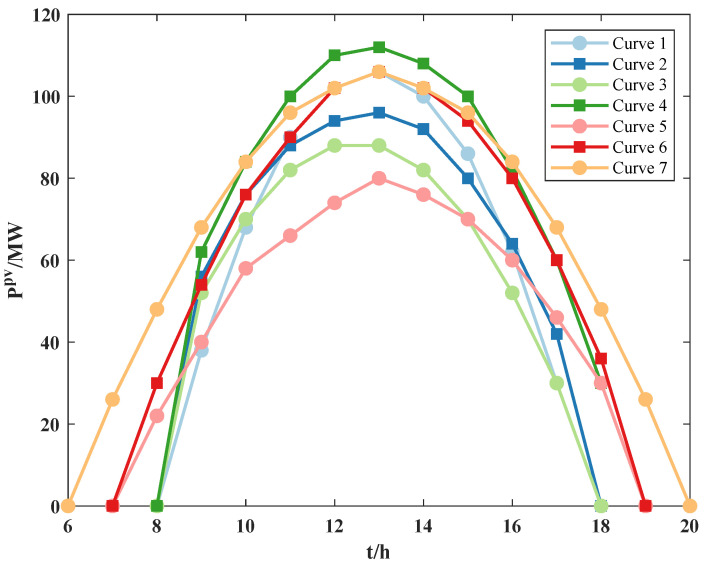
The PV power curve.

Equation (1) indicates that the PV power is weakly correlated with time. There is no significant relationship between PV power at the current moment and the next moment. Therefore, considering the factors that affect PV power in real situations (such as temperature and weather), the relationship between PV power and time is established. Based on the actual PV power data, the relationship is obtained by mathematical fitting, as shown in Figure 2.

It can be seen from Figure 2 that the relationship between PV power and time satisfies the mathematical equation: $P^{pv}=at^{2}+bt+c$. After processing the data, the curve makes it much easier to establish a mathematical model, and curve 1 is equal to test day 1, curve 2 is equal to test day 2, and so on. The time is referred to the Beijing time (BJT), which is eight hours later than Universal Time Coordinated (UTC). The reason is that the solar irradiance is increasing from 6 h to 12 h, the solar irradiance in per unit area is increasing, so the PV power is increasing. At 12–15 h, the solar irradiance basically remains unchanged; the PV power may fluctuate due to the influence of temperature and the power generation efficiency of the solar panel. Then, it generally reaches its maximum value at 13–15 h. After 15 h, the solar irradiance is the main factor while it keeps decreasing. According to Equation (1), the PV power keeps decreasing and decays to 0 at 18–20 h.

To select the best fitting curve, the relevant index ($R^{2}$) and the Nash–Sutcliffe efficiency (NSE) are used as the evaluation criterion to evaluate the accuracy of the fitting curves [24]. The closer values of $R^{2}$ and NSE are to 1, the better the curve is fitted. The fitting accuracy of the curves is shown in Table 1.

Among all the fitting curves, the values of $R^{2}$ and NSE for curve 1 are both closest to 1, which indicates that curve 1 is the best fitting curve. Therefore, this curve is chosen as the fitted PV power curve, and the maximum value of the curve is $P_{rt}^{pv}$.

### 2.2. The Model of PV Power Error

**Figure 3:**
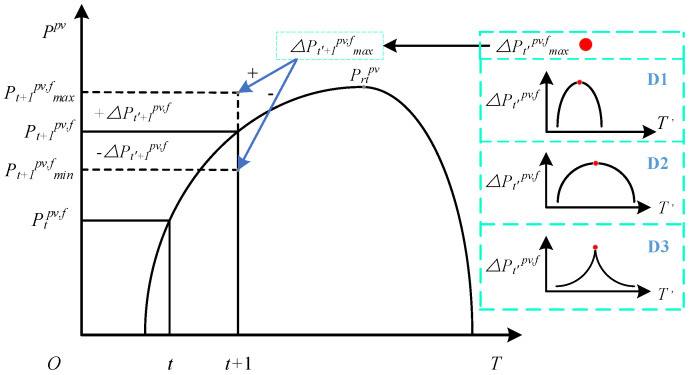
The actual PV power curve.

In this paper, the actual PV power is modeled with reference to the idea of short-term load forecasting, and the PV power uncertainty is expressed as the error obeying a specific distribution function. Then, the actual PV power can be approximated as Equation (2).

(2) $$P_{t}^{pv}=P_{t}^{pv,f}+\Delta P_{t^{\prime }}^{pv,f}$$

where $P_{t}^{pv}$ is the actual PV power at time *t*, $P_{t}^{pv,f}$ is the fitted PV power at time *t*, $\Delta P_{t^{\prime }}^{pv,f}$ is the PV power error at error time $t^{\prime }$, which is considered to obey the Gaussian distribution (D1), Cauchy distribution (D2), and Laplace distribution (D3). The relationship between the actual PV power at time *t* and time t+1 is shown in Figure 3. The red points represent the maximum value of the distribution function. The dashed line indicates the range of the actual PV power at time t+1 ($P_{t+1}^{pv,f}$).

According to Equation (2), the actual PV power is made up of the fitted PV power and the PV power error, the fitted PV power and the PV power error are calculated differently, *t* is used to calculate the fitted PV power, and $t^{\prime }$ is used to calculate the PV power error; then *t* and $t^{\prime }$ are different. With reference to the idea of obtaining the PV power through mathematical fitting, we believe that the distribution of the PV power error should obey a specific pattern based on the fitted PV power, so we use error time $t^{\prime }$ as the x-axis, and the PV power error as the y-axis, so the distribution is the time function.

Where $P_{t}^{pv,f}$, $P_{t+1}^{pv,f}$ are the fitted PV power at time *t* and t+1, and the maximum value of the PV power error at time $t^{\prime }+1$ is

(3) $$\max\Delta P_{t^{\prime }+1}^{pv,f}=0.2\left(P_{t+1}^{pv,f}-P_{t}^{pv,f}\right)$$

Then the maximum and minimum values of $P_{t+1}^{pv}$ at time t+1 are

(4) $$\max P_{t+1}^{pv}=P_{t+1}^{pv,f}+\max\Delta P_{t^{\prime }+1}^{pv,f}$$

(5) $$\min P_{t+1}^{pv}=P_{t+1}^{pv,f}-\max\Delta P_{t^{\prime }+1}^{pv,f}$$

Considering the PV power uncertainty, $P_{t+1}^{pv}$ is

(6) $$P_{t+1}^{pv}=P_{t+1}^{pv,f}+\Delta P_{t^{\prime }+1}^{pv,f}$$

The values of $\Delta P_{t^{\prime }+1}^{pv,f}$ are determined by three distribution functions, and the range of $\Delta P_{t^{\prime }+1}^{pv,f}$ is

(7) $$0\leq \Delta P_{t^{\prime }+1}^{pv,f}\leq \max\Delta P_{t^{\prime }+1}^{pv,f}$$

In summary, the paper establishes the connection between the PV power at time *t* and t+1 using the fitting curves; then, the PV power errors that obey three distribution functions are introduced to simulate the PV power uncertainty. Using the fitting curves and the PV power errors, the model of the PV power uncertainty is established.

## 3. Model of Line-Switch State Uncertainty

**Figure 4:**
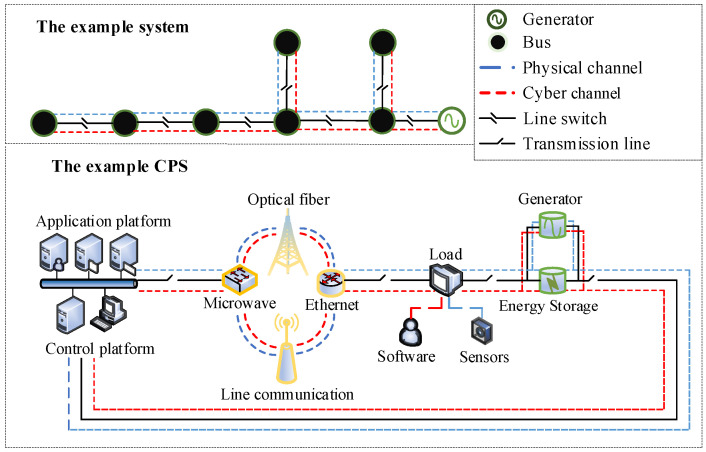
The example bus system and CPS.

As shown in Figure 4, the distribution network can be abstracted as the bus system consisting of buses, generators, and line switches. The line switch mainly plays the role of connections, and its status information is mainly controlled by cyber channel and physical channel. Moreover, through the operations of line switches, transmission lines can be controlled.

However, the actual distribution network can be regarded as the cyber-physical system (CPS) formed by the combination of the physical system and the cyber system. The physical system includes circuit breakers, disconnect switches, interconnection switches, transmission lines, loads, generators, and energy storage units to realize the collection and transmission of electrical data. The cyber system includes application platform, control platform, operation software, and communication technology to realize remote coordination and control of the physical system.

The relationship between the bus system and the CPS is established through the cyber channel and the physical channel: switches and transmission lines in the CPS constitute the main body of the physical channel in the bus system, while software and communication technology in the CPS constitute the main body of the cyber channel in the bus system. The load in the CPS can be simplified as the bus in the bus system; the sensors in the load are used to collect voltage and current information from electricity consumers; the software in the load is used to transmit the digital information; the generator in the CPS can be simplified as a PV generator and system generator in the bus system; the optical fiber and microwave, ethernet, and line communication in the CPS can be simplified as the main part of the cyber channel and physical channel in the bus system (their hardware devices are used to collect and transmit data, and their communication devices are used to receive and execute commands); the circuit breakers, disconnect switches, interconnection switches in the CPS can be incorporated into the line switch in the bus system, and the application platform and control platform in the CPS can be simplified as the schedule center in the bus system. For example, in the CPS, when the power consumption behavior occurs at the load, sensors collect and obtain the voltage information; then, the software transmits it to the application platform and the control platform through the optical fiber. The control platform is mainly used to analyze whether this power consumption behavior is normal or not. If the power consumption behavior is abnormal, the application platform issues a power outage command, which is transmitted to the load through the optical fiber. During the process, the circuit breaker can be shut down directly, or this command can realize the shutdown operation through the load’s software; both of them can achieve the purpose of power-off. In the bus system, the power consumption behavior is generated at the bus; through the line switch, the information is transmitted to the schedule center, which will analyze whether the behavior is normal or not. If the behavior is abnormal, the schedule center issues a disconnect command, then the line switch is disconnected, thus achieving the purpose of power-off at the bus.

When the actual distribution network works normally, the schedule center controls transmission lines, generators, and loads through the cyber system based on the information collected by the physical system, and transmission lines, generators, and loads can send information to schedule center and receive its instructions. At this point, the status of line switches in the bus system are normal.

When faults occur in the actual distribution network, the schedule center completes load transfer and tide equalization while considering physical system failures and cyber system failures. At this point, due to the physical equipment failure, communication transmission failure, and scheduling control failure, the line-switch status uncertainty in the bus system occurs, resulting in the transmission line failure. We assume that faults in the bus system only occur in the transmission line, and the uncertainty of the line-switch status is the cause of the transmission line fault.

### 3.1. The Physical Equipment Failure

The actual distribution network contains massive physical equipment, which is composed of the basic components for collecting electrical information. Once such equipment fails, the command from the schedule center will not be executed; while the schedule center cannot be informed of the status information of the equipment, this situation is considered as a form of the line-switch status uncertainty in the bus system. For example, the schedule center needs the designated disconnect switches to be closed, but some of the designated disconnect switches cannot be connected to transmission lines. After analyzing the power flow, schedule center can accurately know the overall status information of the designated disconnect switches, but the status information of faulty disconnect switches cannot be known.

Then, considering the physical equipment failure, the probability of state uncertainty in line switches is:

(8) $$p_{phy}=w_{e1}\left\{1-\left(1-p^{eb}\right)\left(1-p^{ec}\right)\left(1-p^{es}\right)\right\}+w_{e2}\left\{1-\left(1-p^{ea}\right)\left(1-p^{er}\right)\right\}$$

where $p_{phy}$ represents the probability of physical equipment failure, $w_{e1}$ represents the weight when transmission lines perform abnormal operations, $p_{eb}$ represents the probability when circuit breakers perform abnormal operations, $p_{ec}$ represents the probability when interconnection switches perform abnormal operations, $p_{es}$ represents the probability when disconnect switches perform abnormal operations, $w_{e2}$ represents weight when physical components perform abnormal operations, $p_{ea}$ represents the probability when physical components perform abnormal operations, and $p_{er}$ represents the probability of physical components failure.

In this paper, the line-switch status uncertainty includes rejection and maloperation. When the schedule center designates the line switch to be closed, the line switch refuses to execute the action; it is manifested as rejection, resulting in the transmission line fault. When the schedule center designates the line switch to be disconnected, the line switch refuses to execute the action, and the transmission line is closed,; this is manifested as maloperation.

### 3.2. The Communication Transmission Failure

In the actual distribution network, multiple communication technologies and protocols cannot be separated from the information link, and its connectivity is directly related to the reliability of information transmission. Once the connectivity of the information link is lost, the schedule center cannot be informed of the status information of physical equipment, the commands issued by the schedule center cannot be received by physical equipment, or the status information of the physical equipment cannot be transmitted; this situation is considered as a form of the line-switch status uncertainty in the bus system. For example, the electrical energy data collected by physical equipment needs to be transmitted through circuit breakers, interconnection switches, and disconnect switches to the schedule center. During the process, there are four information links. If anyone of the information link loses the connectivity, the schedule center will not be able to know the power flow information of the branch accurately, or the schedule center will not be able to adjust the power flow through operations.

Then, considering the communication transmission failure, the probability of state uncertainty in line switches is

(9) $$p_{link}=pl_{1}*pl_{2}*\cdots *pl_{n}$$

where $p_{link}$ represents the probability of communication transmission failure; $p_{link}$ = 0 indicates that the communication transmission failure has occurred; $p_{link}$ = 1 indicates that the communication transmission failure has not occurred; $p_{li}$ represents the state of the information component; $p_{li}$ = 0 indicates that the information link is not connected; $p_{li}$ = 1 indicates that the information link is connected; and *n* represents the number of information links.

### 3.3. The Scheduling Control Failure

During the transmission progress of the schedule center’s commands in the information link (the bit error), delayed execution may occur. This will result in inconsistency when the commands are executed, and it is considered as a form of the line-switch status uncertainty in the bus system. For example, when commands are transmitted in the information link, commands are continuously disturbed by noise, resulting in the signals not being completely and accurately demodulated when the commands are executed. Then, the schedule center is unable to achieve precise control of lines and components.

That is, the probability of state uncertainty in line switches is

(10) $$p_{control}=p_{bite}p_{delay}$$

where $p_{control}$ represents the probability of scheduling control failure, $p_{bite}$ represents the probability of bit error in information link, and $p_{delay}$ represents the probability of delayed execution in information link. In this paper, the software failure is considered as a form of status information failure, and $p_{bite}$ and $p_{delay}$ are 1 in all information links in this situation.

Above all, the probability of line-switch state uncertainty ($p_{r}$) is

(11) $$p_{r}=w_{p1}p_{phy}+w_{p2}p_{link}+w_{p3}p_{control}$$

where $w_{p1}$, $w_{p2}$, and $w_{p3}$ are the normalized weights of $p_{phy}$, $p_{link}$, and $p_{control}$. As shown in Equation (11), $p_{r}$ differs under different combinations of $w_{p1}$, $w_{p2}$, $w_{p3}$, $p_{phy}$, $p_{link}$, and $p_{control}$, which constitute the probability distribution of $p_{r}$ under multiple scenarios.

## 4. The Model

### 4.1. The Proposed Model

We propose a multi-objective two-level planning model in this section; the objective is to minimize the maintenance cost under the worst-case condition, and we assume that the line switches are the only components that can be affected in the bus syetem. First, we consider the uncertainty of PV power, and we determine the set of PV power by means of fitting. Then, we consider the uncertainty of the line-switch state under multiple scenarios, which is determined through entropy. Finally, we treat the maximum power cost and the maximum failure cost as the worst-case condition, and we aim to minimize the maintenance cost by simulating commands from the schedule center. The nomenclature is shown in Appendix A.

#### 4.1.1. The Objective Function

The objective function (12) is to minimize the maintenance cost under the worst case. Respectively, the maintenance cost, the power cost, and the failure cost are represented by Equations (13)–(16).

(12) $$f=\min C_{cons}\left\{\max C_{aloss}\right\}$$

(13) $$C_{cons}=\sum_{ij\in \Omega_{l}}c_{ij,t}^{l}\eta_{ij,t}$$

(14) $$C_{aloss}=C_{pvp}+C_{pfom}$$

(15) $$C_{pvp}=\sum_{i\in \Omega_{p}}c_{pv,t}P_{i,t}^{s,pv}$$

(16) $$C_{pfom}=\sum_{ij\in \Omega_{l}}c_{omij,t}^{l}\lambda_{ij,t}^{s}$$

#### 4.1.2. The Power Flow Constraint

Constraints (17)–(20) are linearized DistFlow equations, while constraints (17) and (18) represent the power balance at each bus, and constraints (19) and (20) represent the voltage level at each bus. The DistFlow equations are used to describe the complex power flows at each bus.

(17) $$P_{jk,t}^{s}=P_{ij,t}^{s}-P_{j,t}^{s,d}+\Delta P_{j,t}^{s,d}+P_{j,t}^{s,g}+P_{j,t}^{s,pv}$$

(18) $$Q_{jk,t}^{s}=Q_{ij,t}^{s}-Q_{j,t}^{s,d}+\Delta Q_{j,t}^{s,d}+Q_{j,t}^{s,g}$$

(19) $$-\left(1-\lambda_{ij,t}^{s}\right)M_{1}\leq U_{i,t}^{s}-U_{j,t}^{s}-\frac{P_{ij,t}^{s}r_{ij,t}+Q_{ij,t}^{s}x_{ij,t}}{U_{0}}$$

(20) $$U_{i,t}^{s}-U_{j,t}^{s}-\frac{P_{ij,t}^{s}r_{ij,t}+Q_{ij,t}^{s}x_{ij,t}}{U_{0}}\leq \left(1-\lambda_{ij,t}^{s}\right)M_{1}$$

#### 4.1.3. The PV Power Uncertainty Constraint

Equations (21)–(23) represent the relationship between the PV power and time. Equation (22) assumes that the *j*th PV power at time *t* ($P_{j,t}^{s,pv,ft}$) can be obtained though the fitting curve 1. Equation (23) assumes that the power error of *j*th PV at error time $t^{\prime }$ ($\Delta P_{j,t^{\prime }}^{s,pv,fet}$) can be obtained through the distribution functions.

(21) $$P_{j,t}^{s,pv}=P_{j,t}^{s,pv,ft}+\Delta P_{j,t^{\prime }}^{s,pv,fet}$$

(22) $$P_{j,t}^{s,pv,ft}=\left[P_{j,t}^{s,pv,ft_{1}}\cdots P_{j,t}^{s,pv,ft_{n}}\right]$$

(23) $$\Delta P_{j,t^{\prime }}^{s,pv,fet}=[\Delta P_{j,t^{\prime }}^{s,pv,fet_{1}}\cdots \Delta P_{j,t^{\prime }}^{s,pv,fet_{n}}]\;$$

#### 4.1.4. The Line-Switch State Uncertainty Constraint

Constraints (24) and (25) represent the relationship between the system uncertainty budget and the probability of line-switch state uncertainty. Constraint (24) is related to the Claude Shannon’s information theory, and *W* is the uncertainty budget that can be decided by schedule center. That is, the line state with a higher uncertainty probability takes up less uncertainty budget if failure occurs. Constraint (25) provides the set of the state uncertainty in the line switch, $pr_{ij,t}^{s1}$–$pr_{ij,t}^{s1}$ represent the state uncertainty probability $pr_{ij,t}^{s}$ under multi scenarios. For example, $pr_{ij,t}^{s1}$ represents the scenario that $w_{p1}$ is 1, $p_{phy}$ is 0.7, $w_{p2}$ is 0, $p_{link}$ is 1, $w_{p3}$ is 0, $p_{control}$ is 0.5, and $pr_{ij,t}^{s}$ is mainly decided by the physical device failure.

(24) $$\sum_{ij\in \Omega_{l}}-\log_{2}\beta_{ij,t}^{s}pr_{ij,t}^{s}\leq W\;$$

(25) $$pr_{ij,t}^{s}=\left[pr_{ij,t}^{s_{1}}\cdots pr_{ij,t}^{s_{n}}\right]\;$$

#### 4.1.5. The Line State Constraints

Constraints (26)–(32) represent the relationship between $\alpha_{ij,t}^{s}$, $\beta_{ij,t}^{s}$, $\lambda_{ij,t}^{s}$, and $\eta_{ij,t}^{s}$. For example, $\alpha_{ij,t}^{s}$ = 0, the line ij is closed by the schedule center. Under this condition, if the state uncertainty occurs in the line switch ($\beta_{ij,t}^{s}$ = 1), the actual state of line ij is closed ($\lambda_{ij,t}^{s}$ = 1), and line ij needs to be maintained. If $\beta_{ij,t}^{s}$ = 0, the actual state of line ij ($\lambda_{ij,t}^{s}$ = 0) is broken, and line ij also needs to be maintained. $w_{ij,t}^{s}$ is used to linearize the relationship between $\alpha_{ij,t}^{s}$, $\beta_{ij,t}^{s}$, and $\lambda_{ij,t}^{s}$. Through constraints (26)–(32), the connections of the maintenance cost, the power cost, and the failure cost are established.

(26) $$\alpha_{ij,t}^{s}+\beta_{ij,t}^{s}\leq 2$$

(27) $$\alpha_{ij,t}^{s}+\lambda_{ij,t}^{s}\leq 2$$

(28) $$\beta_{ij,t}^{s}+\lambda_{ij,t}^{s}\leq 2$$

(29) $$\alpha_{ij,t}^{s}\leq \eta_{ij,t}^{s}$$

(30) $$\beta_{ij,t}^{s}\leq \eta_{ij,t}^{s}$$

(31) $$\lambda_{ij,t}^{s}\leq \eta_{ij,t}^{s}\;$$

(32) $$\alpha_{ij,t}^{s}+\beta_{ij,t}^{s}+\lambda_{ij,t}^{s}+2w_{ij,t}^{s}=2$$

#### 4.1.6. The Voltage and Power Constraints

Constraints (33) and (34) limit the active and reactive power flow of line ij. Constraints (35) and (36) limit the active and reactive power of generators. Constraint (37) imposes the voltage limits. Constraints (38) and (39) limit active and reactive load shedding.

(33) $$-\lambda_{ij,t}^{s}P_{ij}^{\max}\leq P_{ij,t}\leq \lambda_{ij,t}^{s}P_{ij}^{\max}$$

(34) $$-\lambda_{ij,t}^{s}Q_{ij}^{\max}\leq Q_{ij,t}\leq \lambda_{ij,t}^{s}Q_{ij}^{\max}$$

(35) $$P_{g}^{\min}\leq P_{j,t}^{s,g}\leq P_{g}^{\max}$$

(36) $$Q_{g}^{\min}\leq Q_{j,t}^{s,g}\leq Q_{g}^{\max}$$

(37) $$U_{j}^{\min}\leq U_{j,t}^{s}\leq U_{j}^{\max}$$

(38) $$0\leq \Delta P_{j,t}^{d}\leq P_{j}^{d\max}$$

(39) $$0\leq \Delta Q_{j,t}^{d}\leq Q_{j}^{d\max}$$

#### 4.1.7. The Islanding Constraint

Constraints (40)–(42) guarantee the radiality topology of the distribution network when the state uncertainty occurs in the line switch. Constraint (40) guarantees that the number of buses and the number of lines conform to the radial constraint. Based on the directed multicommodity flow model, constraint (41) supposes all buses that exclude the fictitious bus have 1 unit of load demand, so that the connectivity of the distribution network is guaranteed. Constraint (42) limits the fictitious power flow of line ij. Particularly, the virtual lines are determined by virtual buses and buses of broken lines. When we only consider the PV power uncertainty, as the PV power becomes smaller, the load demand decreases, and the shedding load increases; the load demand at the bus may be completely removed because the distribution network reconfiguration is not considered. When we consider the case of considering PV source and switches as uncertain, we ensure that the radial topology remains unchanged during the distribution network reconfiguration, and this is more relevant to the actual situation.

(40) $$\sum_{ij\in \Omega_{l\_vir}}\mu_{ij,t}^{s}=n_{b}-1\;ij\in \Omega_{l\_vir}$$

(41) $$\sum_{ji\in \Omega_{l}}f_{ji,t}^{s}-\sum_{ij\in \Omega_{l}}f_{ij,t}^{s}=1\;ij\in \Omega_{l\_vir}$$

(42) $$-\mu_{ij,t}^{s}M_{2}\leq f_{ji,t}^{s}\leq \mu_{ij,t}^{s}M_{2}\;ij\in \Omega_{l\_vir}$$

### 4.2. Solution Algorithm

In the above formulation, $P_{j,t}^{s,pv}$ represents the variables of the power cost, $\beta_{ij,t}^{s}$ represents the variables of the failure cost, and $\eta_{ij,t}^{s}$ represents the variables of the maintenance cost. We solve the min–max problem by expanding the problem to the min–max–max problem. That is, we take $P_{j,t}^{s,pv}$ as the first-level variable to maximize the power cost, we take $\beta_{ij,t}^{s}$ as the second-level variable to maximize the failure cost, and we take $\eta_{ij,t}^{s}$ as the third-level variable to minimize the maintenance cost.

The main part of first-level problem can be introduced as follows:

(43) $$\max\sum_{i\in \Omega_{p}}c_{pv,t}P_{i,t}^{s,pv}$$

(44) $$P_{jk,t}^{s}=P_{ij,t}^{s}-P_{j,t}^{s,d}+\Delta P_{j,t}^{s,d}+P_{j,t}^{s,g}+P_{j,t}^{s,pv}$$

(45) $$Q_{jk,t}^{s}=Q{}_{ij,t}^{s}-Q_{j,t}^{s,d}+\Delta Q_{j,t}^{s,d}+Q_{j,t}^{s,g}$$

(46) $$U_{j,t}^{s}=U_{i,t}^{s}-\frac{P_{ij,t}^{s}r_{ij,t}+Q_{ij,t}^{s}x_{ij,t}}{U_{0}}$$

(47) $$P_{j,t}^{s,pv}=P_{j,t}^{s,pv,ft}+\Delta P_{j,t^{\prime }}^{s,pv,fet}$$

(48) $$P_{j,t}^{s,pv,ft}=\left[P_{j,t}^{s,pv,ft_{1}}\cdots P_{j,t}^{s,pv,ft_{n}}\right]$$

(49) $$\Delta P_{j,t^{\prime }}^{s,pv,fet}=[\Delta P_{j,t^{\prime }}^{s,pv,fet_{1}}\cdots \Delta P_{j,t^{\prime }}^{s,pv,fet_{n}}]$$

Given a series of sets of $P_{j,t}^{s,pv}$, we can determine the time set when the power cost of PV is maximum. Moreover, we analyze the impact of the power error on the cost by its fluctuation.

The main part of second-level problem can be introduced as follows:

(50) $$\max\sum_{ij\in \Omega_{l}}c_{omij,t}^{l}\lambda_{ij,t}^{s}$$

(51) $$P_{jk,t}^{s}=P_{ij,t}^{s}-P_{j,t}^{s,d}+\Delta P_{j,t}^{s,d}+P_{j,t}^{s,g}+P_{j,t}^{s,pv}$$

(52) $$Q_{jk,t}^{s}=Q{}_{ij,t}^{s}-Q_{j,t}^{s,d}+\Delta Q_{j,t}^{s,d}+Q_{j,t}^{s,g}$$

(53) $$-\left(1-\lambda_{ij,t}^{s}\right)M_{1}\leq U_{i,t}^{s}-U_{j,t}^{s}-\frac{P_{ij,t}^{s}r_{ij,t}+Q_{ij,t}^{s}x_{ij,t}}{U_{0}}$$

(54) $$U_{i,t}^{s}-U_{j,t}^{s}-\frac{P_{ij,t}^{s}r_{ij,t}+Q_{ij,t}^{s}x_{ij,t}}{U_{0}}\leq \left(1-\lambda_{ij,t}^{s}\right)M_{1}$$

(55) $$\sum_{ij\in \Omega_{l}}-\log_{2}\beta_{ij,t}^{s}pr_{ij,t}^{s}\leq W$$

(56) $$pr_{ij,t}^{s}=\left[pr_{ij,t}^{s_{1}}\cdots pr_{ij,t}^{s_{n}}\right]$$

(57) $$\sum_{ij\in \Omega_{l\_vir}}\mu_{ij,t}^{s}=n_{b}-1\;ij\in \Omega_{l\_vir}$$

(58) $$\sum_{ji\in \Omega_{l}}f_{ji,t}^{s}-\sum_{ij\in \Omega_{l}}f_{ij,t}^{s}=1\;ij\in \Omega_{l\_vir}$$

(59) $$-\mu_{ij,t}^{s}M_{2}\leq f_{ji,t}^{s}\leq \mu_{ij,t}^{s}M_{2}\;ij\in \Omega_{l\_vir}$$

When state uncertainty occurs, the islanding effect maybe appear in the distribution network, so it is necessary to introduce virtual buses to ensure that the distribution network remains the radial topology after reconfiguration. Furthermore, we describe the randomness of state uncertainty probabilities in multiple scenarios by using the Claude Shannon’s information theory, and we use the uncertainty budget to simplify the process of selecting scenarios.

The main part of the third-level problem can be introduced as follows:

(60) $$\min\sum_{ij\in \Omega_{l}}c_{ij,t}^{l}\eta_{ij,t}$$

(61) $$P_{jk,t}^{s}=P_{ij,t}^{s}-P_{j,t}^{s,d}+\Delta P_{j,t}^{s,d}+P_{j,t}^{s,g}+P_{j,t}^{s,pv}$$

(62) $$Q_{jk,t}^{s}=Q{}_{ij,t}^{s}-Q_{j,t}^{s,d}+\Delta Q_{j,t}^{s,d}+Q_{j,t}^{s,g}$$

(63) $$-\left(1-\lambda_{ij,t}^{s}\right)M_{1}\leq U_{i,t}^{s}-U_{j,t}^{s}-\frac{P_{ij,t}^{s}r_{ij,t}+Q_{ij,t}^{s}x_{ij,t}}{U_{0}}$$

(64) $$U_{i,t}^{s}-U_{j,t}^{s}-\frac{P_{ij,t}^{s}r_{ij,t}+Q_{ij,t}^{s}x_{ij,t}}{U_{0}}\leq \left(1-\lambda_{ij,t}^{s}\right)M_{1}$$

(65) $$\alpha_{ij,t}^{s}+\beta_{ij,t}^{s}\leq 2$$

(66) $$\alpha_{ij,t}^{s}+\lambda_{ij,t}^{s}\leq 2$$

(67) $$\beta_{ij,t}^{s}+\lambda_{ij,t}^{s}\leq 2$$

(68) $$\alpha_{ij,t}^{s}\leq \eta_{ij,t}^{s}$$

(69) $$\beta_{ij,t}^{s}\leq \eta_{ij,t}^{s}$$

(70) $$\lambda_{ij,t}^{s}\leq \eta_{ij,t}^{s}$$

(71) $$\alpha_{ij,t}^{s}+\beta_{ij,t}^{s}+\lambda_{ij,t}^{s}+2w_{ij,t}^{s}=2$$

In this model, we consider the worst-case scenario: the presence of islands in the distribution network leads to the fragmentation of its network structure. If the line is not maintained, all the load demand of the buses will be removed. Therefore, it is necessary to consider how to achieve line control by the schedule center with minimal maintenance costs. According to the first-level problem and the second-level problem, the PV power and line states are known, so we can obtain the optimal control strategy by constraints (60)–(71) to provide a reference basis for system planners.

Next, we present the implementation steps of our algorithm; we use the Latin hypercube sampling (LHS) method to determine the state when the uncertainty budget is different. The LHS method improves the sampling strategy to achieve a higher sampling accuracy with a smaller sampling size, and its sampling results are closer to the actual situation.

Step 1 sets the lower bound of time $LB_{t}$ = 0, the upper bound of time $UB_{t}$ = 24, solves the first-level problem (43)–(49), obtains its optimal value, and updates $LB_{t}$ and $UB_{t}$ with the optimal value; then, it obtains the lower bound of PV power $LB_{pvt}$ and the upper bound of PV power $UB_{pvt}$.

Step 2 sets the uncertainty budget and the number of the line-switch state, obtains its optimal value using LHS method, and updatse $LB_{p}v$ and $UB_{p}v$ with the optimal value.

Step 3 is based on the optimal PV power and the optimal line-switch state; it solves the third-level problem (60)–(71) and obtains its optimal value.

## 5. Numerical Results

**Figure 5:**
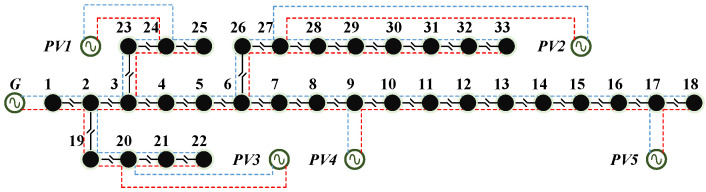
IEEE 33-bus distribution system.

As shown in Figure 5, we use the modified IEEE 33-bus system for simulation analysis. For illustration, there are 33 buses, 32 lines, 1 system generator, and 5 PV generators in the test system, and the reference voltage is 12.66 kV. The system generator is placed at bus 1; the PV generators are placed at bus 9, 17, 20, 24, 27; and their capacities are 100 MW, 110 MW, 120 MW, 130 MW, and 140 MW. We set the failure cost of the one-switch to 1 $ ($c_{omij,t}^{l}$ = 1 $) and the maintenance cost of one-line to 1 $ ($c_{ij,t}^{l}$ = 1 $). We believe that data in the distribution network can be affected by the cyber system, causing us to receive the wrong data, even if these data are correct in the collection phase. Furthermore, the scenarios we set are considered beforehand.

As for the power cost, we establish the change law of PV power under actual conditions in Section 2, so in this section we propose to determine the power cost under actual conditions. For illustration, we consider the mathematical equation of $c_{pv,t}$ and $t_{sum}$ as

(72) $$c_{pv,t}=at_{sum}^{2}+bt_{sum}+c$$

where *a*, *b*, *c* represents the coefficient of the equation, and $t_{sum}$ represents the accumulated time of PV power. We take 6 h as the starting time $t_{ini}$ and determine the generation time *t* according to curve 1; the accumulated time is $t_{sum}$ = *t* − $t_{ini}$. Furthermore, we set the time step of PV power as 1 h and the time period as 24 h.

The model proposed in this paper is programmed by using MATLAB and solved by using the commercial solver GUROBI. The configuration of the computer is CPU: i7-9700, RAM: 32GB, and 2666 MHz.

### 5.1. The Output Cost of PV

In Section 2, we use the fitting approach to determine the PV power while we consider the actual situation, and we propose to introduce the power error to simulate the uncertainty of the PV power. However, the power error obeying different distributions may have an impact on the PV power and thus on the power cost. Therefore, it is necessary to study the impact of different error distributions to obtain the maximum cost of PV power.

**Figure 6:**
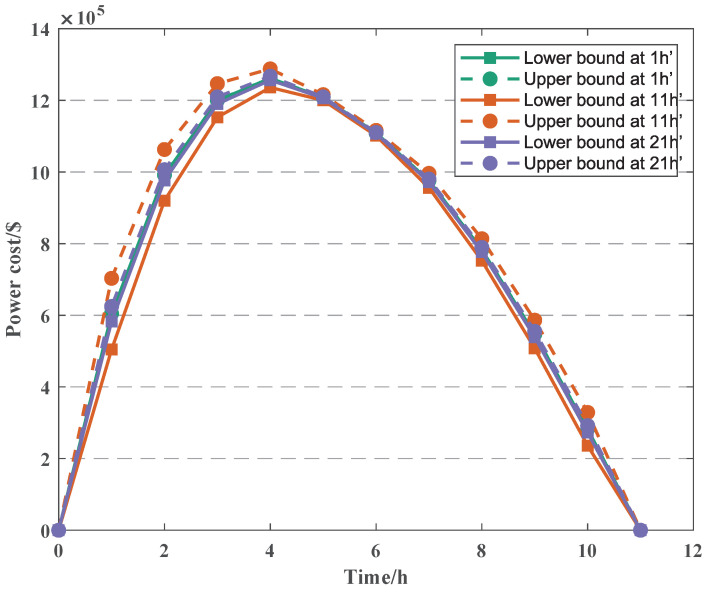
The cost with different power error.

We analyze the overall impact of the power error on the power cost, and we set the error time at 1 h’, 11 h’, and 21 h’, as shown in Figure 6 (the power error follows the Gaussian distribution).

As can be seen from the figure, the power cost and the PV power are basically consistent, while the power cost reaches the maximum at 11 h, and the PV power reaches the maximum at 15 h. The reason for this situation is that we consider the relationship between accumulated time ($t_{sum}$) and power cost ($c_{pv,t}$) in the actual situation; the shorter the accumulated time, the higher the power cost (the power cost is 2.3991 $/W at 11 h, and the power cost is 1.7081 $/W at 15 h), which indicates that the accumulated time $t_{sum}$ has a greater impact on the power cost $c_{pv,t}$.

Furthermore, we can know that the fluctuation of the power error has the greatest impact on the power cost when $t^{\prime }$ is 11 h, followed by 21 h; when $t^{\prime }$ is 1 h, the upper bound and lower bound of the power cost are almost the same. The reason for this situation is that we assume the PV power error to vary with error time ($t^{\prime }$). According to the Gaussian distribution, the power error is the largest when $t^{\prime }$ is 11 h; the PV power fluctuates the most, so the difference between the upper bound and lower bound of the cost can be obviously observed at $t^{\prime }$ = 11 h. Additionally, when $t^{\prime }$ = 1 h, the PV power error is the smallest, resulting in the upper and lower bounds of the power cost almost overlapping. This also indirectly shows that the uncertainty of PV power has an impact on the power cost $c_{pv,t}$.

**Figure 7:**
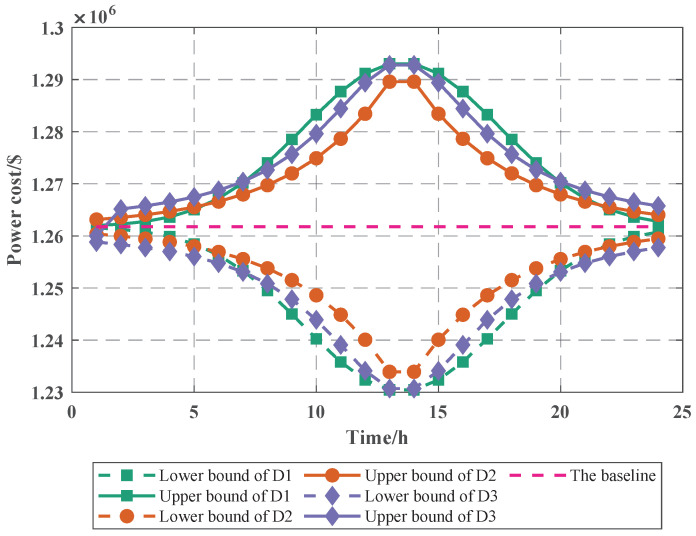
The cost under different distribution.

Based on Figure 6, we synthesize the impact of the power cost when the PV power error follows different distributions. As shown in Figure 7, we find that the impact of PV power error on the power cost remains consistent with the distribution of PV power error, and the baseline of its fluctuation is the curve when PV power error is not considered, and when the error time is 13 h ($t^{\prime }$ = 13 h), the cost all achieves the maximum in three distributions. After considering the PV power error, if the PV power error follows the Gaussian distribution, the upper and lower bounds of the power cost obey the Gaussian distribution.

When the PV power error is considered, the PV power will be taken as the maximum to maximize the power cost; especially in this paper, there is no removal of bus load after considering the system generator. Additionally, since we choose to select the power cost as the first-level variables, the upper and lower bounds of output cost are determined directly by PV power and PV power error; once PV power is determined, the change of power cost is only affected by PV power error. Through the change of PV power error, we can visually analyze the change of power cost, which indicates that the proposed PV power uncertainty model is more intuitive. This also indicates that modifying the maximum value of the PV power error at time $t^{\prime }+1$ does not result in a change in the trend of the output cost.

**Figure 8:**
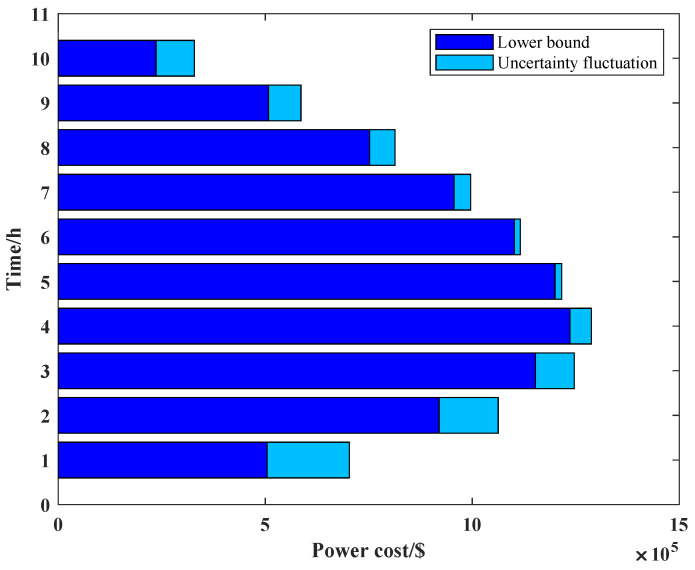
The cost considering uncertainty.

We also analyze the impact of the time on the power cost, as shown in Figure 8 (the error time $t^{\prime }$ is 11 h, and the PV power error follows the Gaussian distribution). The variation of the power cost remains consistent with Figure 6, but the upper and lower bounds of the power cost are more influenced by the time. When the time is 11 h (*t* = 11 h), the lower bound of the power cost is 1.15 M $, while the upper bound is 1.25 M $, an increase of 8.6%, but when the time is 8 h (*t* = 8 h), the lower bound of the power cost is 0.5 M $, and the upper bound is 0.7 M $, an increase of 40%. This shows that as the PV power continues to become larger, the PV power error has less influence on the power cost, and the PV power has a stronger influence.

Through the above analysis, the time plays a major role in the PV power cost, and its change law is consistent with the PV power curve; when the time is 11 h, the power cost is maximum. Once the time is determined, the PV power cost is affected by the PV power error, and it is consistent with the change of the power error; when the error time is 13 h, the power cost is maximum.

### 5.2. The Failure Cost of Line Switches

We simplify the causes of state uncertainty in the actual distribution network in Section 3 and then constitute state uncertainty probability under multiple scenarios by normalizing the weights, and in Section 4 we introduce uncertainty budget for line state selection, so we analyze the impact of the uncertainty budget on the failure cost under different scenarios.

**Figure 9:**
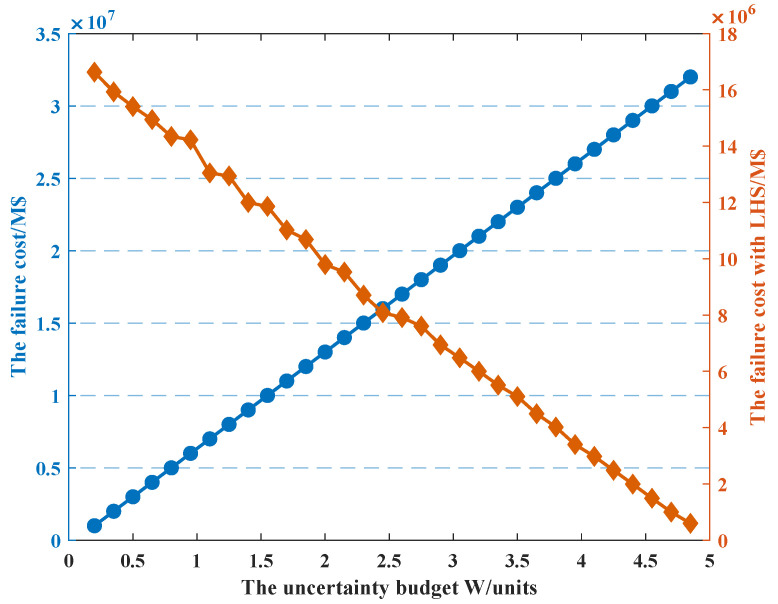
The relationship between W and failure cost.

We consider the relationship between the failure cost and the uncertainty budget as we set $pr_{ij,t}^{s}$ = 0.9. As shown in Figure 9, the failure cost and uncertainty budget *W* satisfy a linear relationship when the LHS method is not used. The larger the uncertainty budget set by the schedule center, if the state failure probability of the line switch is determined, the greater the number of state failures occurring on the line; then, the greater the number of line state abnormalities will result in a larger failure cost.

**Figure 10:**
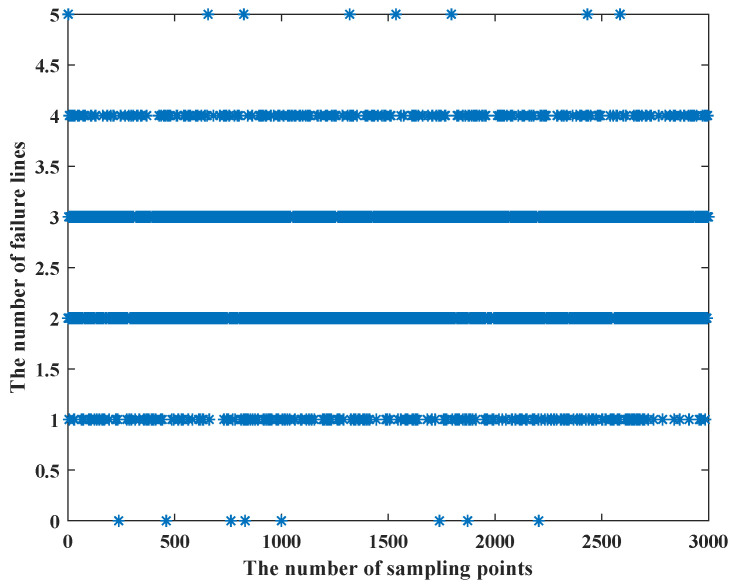
The LHS method process when the number of failure lines is 5.

**Figure 11:**
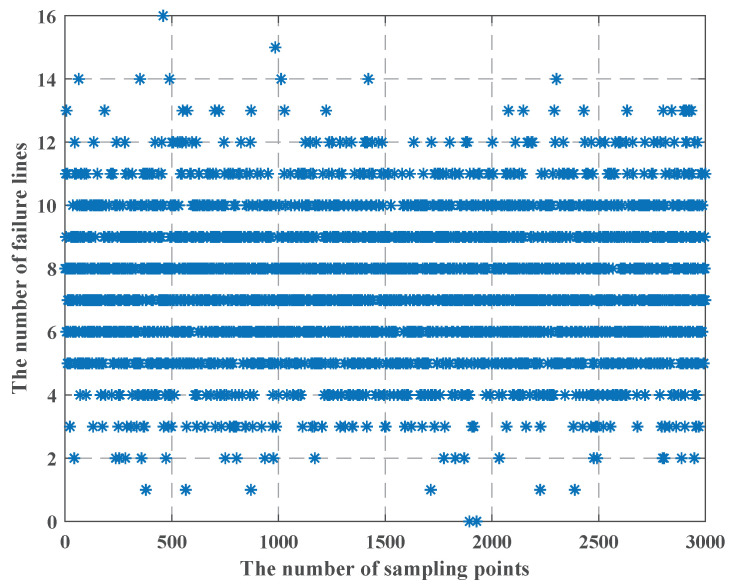
The LHS process method when the number of failure lines is 15.

**Figure 12:**
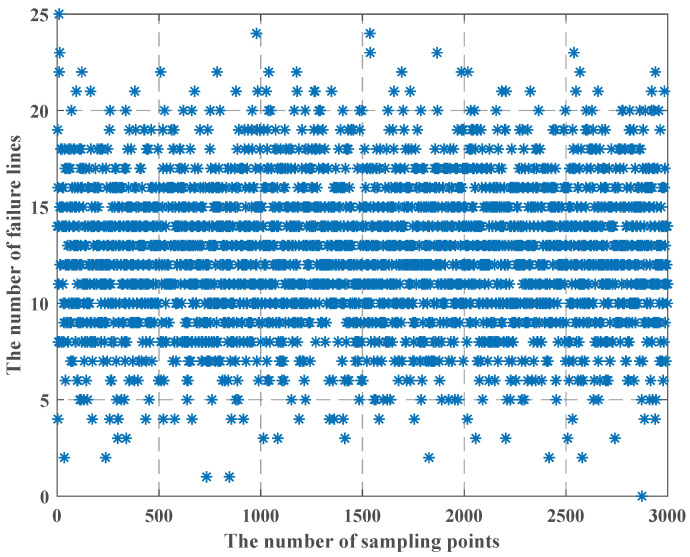
The LHS process method when the number of failure lines is 25.

When we use the LHS method, we find that as the uncertainty budget increases, and the failure cost is less, which is in apparent conflict with constraint (25), so we use Figure 10, Figure 11 and Figure 12 to show the process of the LHS method (we consider the uncertainty probability of line switch to remain unchanged, and we set the uncertainty budget to be 5 units). We find that after we use the LHS method, the distribution of line switches with state uncertainty is reasonable, and it is closer to the real situation: all the lines switches with state uncertainty, and none of line switches with state uncertainty occur the least, while most cases are in between of them.

From Figure 10, Figure 11 and Figure 12, we can see that as the number of failure lines increases, its percentage keeps decreasing. For example, there may occur state uncertainty in five line switches (nf = 5); during the sampling progress, there may be 0, 1, 2, 3, 4, and 5 failure lines; their percentages are 1%, 7%, 42%, 42%, 7%, and 1%. When nf = 15, the percentage of the maximum value is 17%; when nf = 25, the percentage of the maximum value is 11%; when nf = 32, the percentage of the maximum value is 8.4%; and the failure cost is 32 M $, which is consistent with constraint (25). However, the use of the LHS method needs to consider all state of line switches, such as nf = 31, nf = 20, and other cases. Then, according to Figure 9, the failure cost in these cases decreases, combining with its percentage; these will lead to a decay of the failure cost. Similarly, we can vary the uncertainty budget to analyze the impact of different nf.

**Figure 13:**
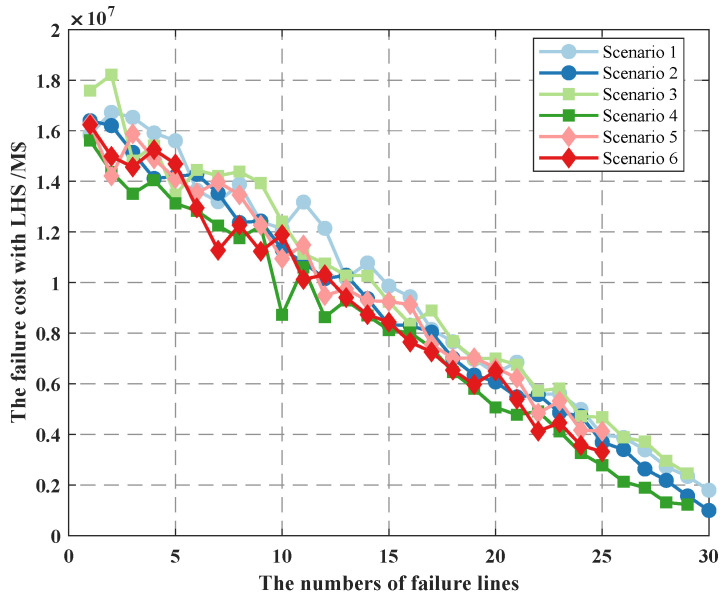
The failure cost under different scenarios.

We also consider the variation of failure cost for different scenarios, as shown in Figure 13. We set the uncertain budget cost to be five units—$p_{phy}$ = 0.9, $p_{link}$ = 1, and $p_{control}$ =0.7—and we adjust the probability by weights to ensure that the range of values for line-switch state information failure is 0.9,0.7,0.5. Scenario 1 represents that all line switches have a probability of 0.9; scenario 2 represents that there are only 2 line switches that have a probability of 0.7, 0.5; scenario 3 represents that there are only 4 line switches that have a probability of 0.7, 0.5; scenario 4 represents that there are only 8 line switches that have a probability of 0.7, 0.5; scenario 5 represents that there are only 10 line switches that have a probability of 0.7, 0.5; and scenario 6 represents that there are only 14 line switches that have a probability of 0.7, 0.5.

As seen in the figure, as more and more lines fail, the failure cost diminshes, which is consistent with Figure 10. Among all lines, the lower the probability of line-switch state uncertainty, the greater the failure cost, for example, comparing scenario 1 and scenario 2, since the probability of line-switch state uncertainty in scenario 2 is 0.9, 0.7, and 0.5, according to constraint (25), when the uncertain budget is set, the number of failure lines is higher in scenario 1, resulting in the sampling value of each variable in the LHS method being larger, which in turn makes the line failure cost in scenario 1 higher than that in scenario 2. This shows that when we consider the probability of line-switch state uncertainty in different scenarios, the higher the probability of failure of all line-switch states, the higher the failure cost.

Through the above analysis, we find that the effect of uncertainty budget on failure cost is consistent with the effect of line-switch state uncertainty probability after we use the LHS method. That is, to obtain the maximum failure cost, we tend to choose a larger uncertainty budget and state uncertainty probability.

### 5.3. The Maintenance Cost Planning by Schedule Center

**Figure 14:**
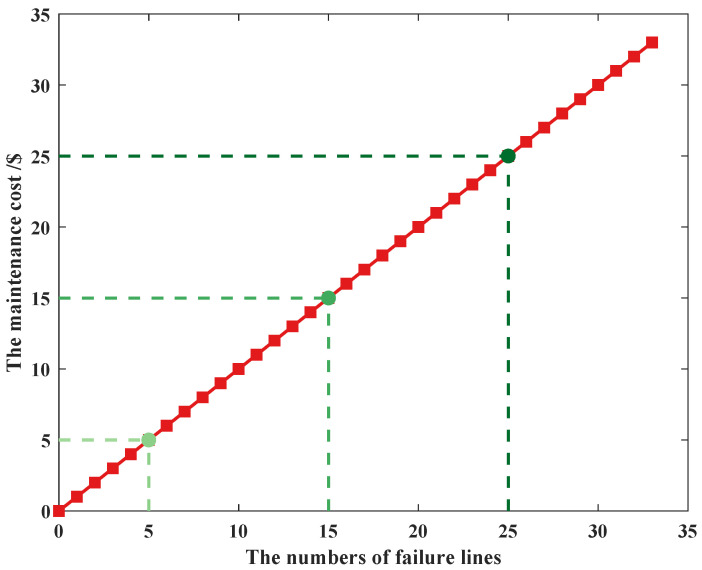
The curve of failure cost.

To minimize the impact caused by the uncertainty of PV power and line-switch status, we aim at using the minimum maintenance cost. The time of PV power is 11 h, the error time is 13 h, the state failure probability is 0.9, and the uncertainty budget is 5 units, as shown in Figure 14.

It shows that the maintenance cost and the number of failure lines satisfy the linear growth relationship; the more failure lines caused by the uncertainty state of line switches, the higher the maintenance cost, and the relationship remains the same when the uncertainty budget is different. For example, if there is only one line switch whose status is not determined ($\beta_{ij,t}^{s}=1$, the line failure occurs), according to constraint (30), the number of lines to be maintained is at least 1 ($\eta_{ij,t}^{s}\leq 1$). To obtain the minimum maintenance cost, the value of $\sum_{ij\in \Omega_{l}}\eta_{ij,t}$ should be small, and $\eta_{ij,t}^{s}$ is supposed to be 1. If the assisted binary variable $w_{ij,t}^{s}$ is 0, then constraint (31) can be simplified as $\alpha_{ij,t}^{s}+\beta_{ij,t}^{s}+\lambda_{ij,t}^{s}=0$, $\beta_{ij,t}^{s}=0$; it conflicts with $\beta_{ij,t}^{s}=1$. Then, $w_{ij,t}^{s}$ is 0, and constraint (31) can be simplified as $\alpha_{ij,t}^{s}+\beta_{ij,t}^{s}+\lambda_{ij,t}^{s}=2$; combined with constraints (26) and (28), it is clear that $\alpha_{ij,t}^{s}\leq 1$, $\lambda_{ij,t}^{s}\leq 1$, according to constraints (29) and (31), $\eta_{ij,t}^{s}=1$. So the maintenance cost remains the same as the number of failure lines.

**Figure 15:**
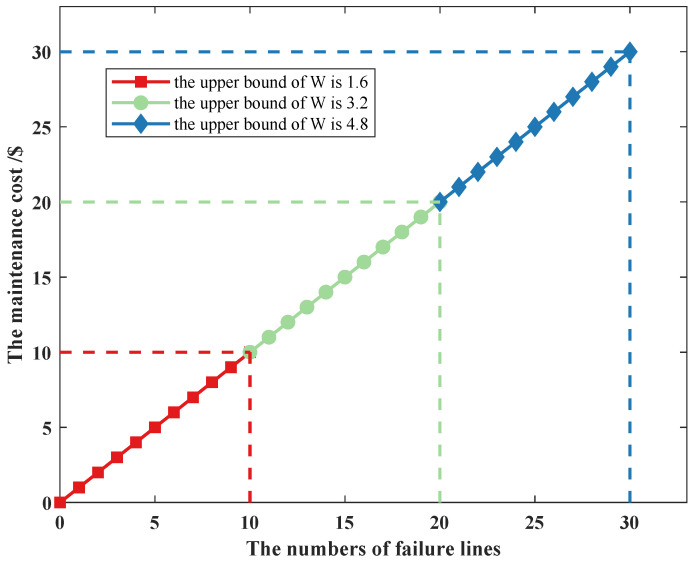
The curve of failure cost considering the upper bound of *W*.

As shown in Figure 15, when we consider the upper bound of uncertainty budget, the maintenance cost still satisfies the linear growth relationship, and it increases as the uncertain budget increases. As we use the Claude Shannon’s information theory, the larger the upper bound of the uncertainty budget, the more frequent the uncertainty of the line-switch status occurs, and the greater the number of line failures, the more lines need to be maintained, leading to an increase in maintenance cost, and the change trend remains consistent with Figure 14. When we consider the change of $pr_{ij,t}^{s}$, the maintenance cost still satisfies the linear growth relationship. According to constraint (24), when *W* remains unchanged, the smaller the value of $pr_{ij,t}^{s}$, the less the number of failure lines, the less the maintenance cost, and the change trend remains consistent with Figure 14. For example, when we consider $pr_{ij,t}^{s}$ of line ij in scenarios 1 to 6, the $pr_{ij,t}^{s}$ in scenario 6 is smaller than the $pr_{ij,t}^{s}$ in scenario 1; according to constraint (24), the uncertainty budget *W* remains unchanged, and the $\beta_{ij,t}^{s}$ in scenario 6 is larger than the $pr_{ij,t}^{s}$ in scenario 1. Thus, the situation is consistent with our analytical approach of considering the upper bound of uncertain budgets. This also shows that when considering line-switch state uncertainty, the probability of its occurrence must be minimized so that maintenance costs can be reduced.

In addition, we analyze the impact of commands on PV power. When the PV power is determined, the actual output of PV power is approximately 76 MW, 86 MW, 90 MW, 100 MW, and 100 MW, which is in consistent with the fitting results. This indicates that as we consider the maintenance cost, the impact caused by the uncertainty of PV power is small. Since the PV will be powered separately after the reconfiguration, its output will be different; the distribution network still mainly relies on the system power. Moreover, the more lines that are shut down by schedule center, the more load needs to be removed from the bus system, which gradually increases from 3.3 MW (the number of failure line is 1) to 13 MW (the number of failure line is 16), which indicates that the schedule center must ensure that no excessive load is removed when shutting down the lines.

**Figure 16:**
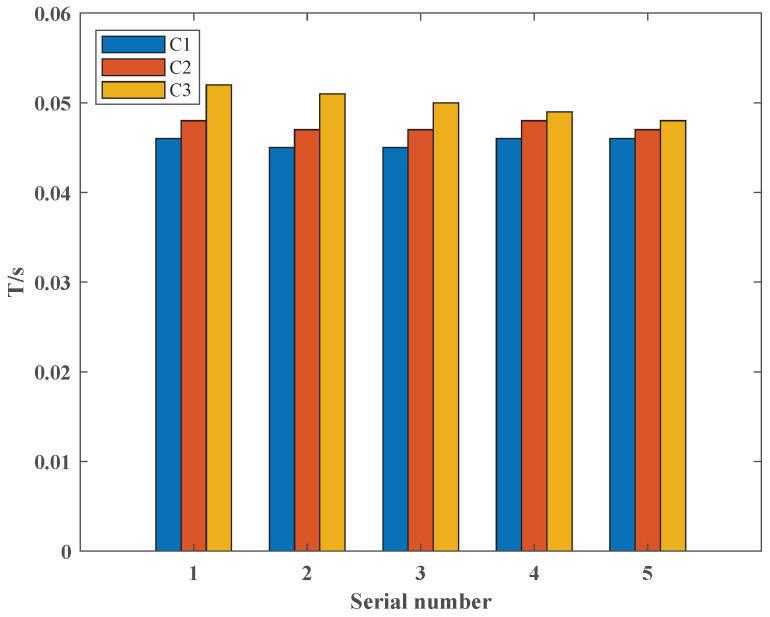
The running time of different experiments.

In order to analyze the advantages of the uncertainty presented in this paper, we compared the running time (*T*); as shown in Figure 16, C1 represents the certainty of PV power, C2 represents the certainty of PV power and line-switch state, and C3 represents the uncertainty in both PV power and line-switch state. We find that the uncertainty presented in this paper has a negligible disadvantage in terms of running time, and the smaller the uncertainty budget *W* is, the shorter the running time is. This is due to the difference in constraints when running the program: the fewer factors considered, the faster the optimization, so the shortest running time is achieved by considering only one factor (PV power or line-switch state). If both factors (PV power and line-switch state) are considered, the increase in constraints leads to an increase in running time. This also shows that for a single simulation experiment, the running time is the same for certainty and uncertainty. Due to the constraints (24) and (25) presented in this paper, additional iterations are required, resulting in an increase in running time. However, as the *W* becomes smaller, the smaller the floating space for uncertainty in PV power and line switching state, i.e., the boundary between uncertainty and certainty becomes blurred, so the running time will be consistent.

**Figure 17:**
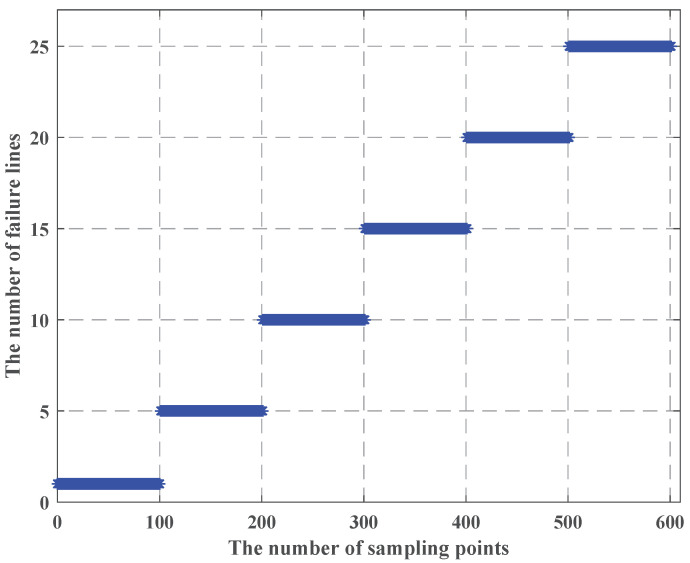
The Monte Carlo method.

We use the Monte Carlo method for sampling, as shown in Figure 17. We find that the number of the sampled failure line is the same, implying that the probability is the same in the actual distribution network. Combined with the analysis of the actual distribution network, the number of failure lines varies with the probability. Furthermore, when carrying out optimization, priority is given to maximizing the objective function, which can also lead to different fault lines having different chances of being sampled. We use the LHS method for uncertainty sampling; Figure 10, Figure 11 and Figure 12 is closer to the actual situation.

Therefore, we believe that the advantages of our proposed uncertainty are as follows: (1) the increase in running time during a single sampling is negligible, which means that when considering many scenarios, we only need to adjust the uncertainty budget *W*, and its running time is basically 0.05 s, which no longer requires repeated iterations and greatly reduces the operation time; (2) the uncertainty sampling results are more realistic after using the LHS method; it can avoid the uniform sampling.

## 6. Conclusions

This paper presents a new approach to protect the distribution networks against the uncertainty of PV power and the line-switch state. The problem is formulated as a multi-objective two-level planning model and then reformulated as a min–max-max model, which is a mixed-integer linear program. The first-level model solves the uncertainty of the PV power problem; its objective is to maximize the PV power cost. The second-level model solves the uncertainty of line-switch state; its objective is to maximize the line failure cost. Lastly, the third-level model solves the maintain problem under the worst-case scenarios; its objective is to minimize the maintenance cost. The proposed model is tested on a modified IEEE 33-bus system. The numerical results show that the proposed model can act as a reference for the schedule center to improve the stability of the distribution network while considering the uncertainty of PV power and the line-switch state.

The major contributions of this paper are as follows:

- We combine mathematical fitting and distribution functions to establish the PV power uncertainty model: we use the relevant index ($R^{2}$) and Nash–Sutcliffe efficiency (NSE) to obtain the best PV fitting curve, and then we use PV power errors that obey three distribution functions to represent the uncertainty of PV power. We have found that once the fitted PV power is determined, the PV power cost is affected by the PV power error, and it is consistent with the change of the distribution functions.
- After conducting an in-depth analysis of the correlation between the bus system and the actual distribution network (CPS), we propose the line-switch state uncertainty model: we guarantee the radial topology during the distribution network reconfiguration process, and we introduce uncertainty budgets and the probability of line-switch state uncertainty, Claude Shannon’s information theory to simplify scenarios, while we use the LHS method for sampling process. We have found that after we use the LHS method, the effect of uncertainty budget on failure cost is consistent with the effect of line-switch state uncertainty probability.
- We propose the maintenance model under the worst-case scenarios: based on the optimal PV power cost and the line failure cost, we have established the linearized line state model to achieve line control by the schedule center. With the minimal maintenance cost, we have found that the maintenance cost and the number of failure lines satisfy the linear growth relationship.

## Figures and Tables

**Table 1:** Comparison of fitting accuracy of each curve.

*Curve*	$R^{2}$	*NSE*
1	0.9605	0.9632
2	0.848	0.9023
3	0.8024	0.7891
4	0.8628	0.8829
5	0.8057	0.8554
6	0.8322	0.8583
7	0.8502	0.709

## Data Availability

Not applicable.

## Abbreviations

The following abbreviations are used in this manuscript:

PV	photovoltaic
DG	distributed generator
LHS	Latin hypercube sampling
CPS	cyber-physical system

## Appendix A. Nomenclature

### Appendix A.1. Sets and Indices

$\Omega_{l\_vir}$: Set of lines containing the fictitious lines.

$\Omega_{l}$: Set of lines.

$\Omega_{b}$: Set of buses.

$\Omega_{t}$: Set of time.

$\Omega_{t^{\prime }}$: Set of error time.

$\Omega_{p}$: Set of photovoltaic generators.

$\Omega_{g}$: Set of generators.

ij: Line index.

*j*: Bus index.

*t*: Time index.

$t^{\prime }$: Error time index.

*s*: Scenario index.

### Appendix A.2. Parameters

*W*: Uncertainty budget.

$c_{ij,t}^{l}$: Maintenance cost of line ij.

$c_{pv,t}$: Output cost of photovoltaic generators.

$c_{omij,t}^{l}$: Failure cost of line switch.

$P_{g}^{\max}$/$Q_{g}^{\max}$: Active/reactive power limits of generators.

$P_{ij}^{\max}$/$Q_{ij}^{\max}$: Active/reactive power limit of line ij.

$P_{j}^{d\max}$/$Q_{j}^{d\max}$: Active/reactive load demand.

$U_{j}^{\max}$/$U_{j}^{\min}$: Maximum/minimum voltage magnitude.

$U_{0}$: Reference voltage magnitude.

$M_{1}$/$M_{2}$: Big numbers.

### Appendix A.3. Variables

$\alpha_{ij,t}^{s}$: Binary variable indicates whether the line ij is closed through the schedule center (1) or not (0).

$\beta_{ij,t}^{s}$: Binary variable indicates whether the line ij is failed due to the state failure of line switch (1) or not (0).

$\lambda_{ij,t}^{s}$: Binary variable indicates whether the actual state of line ij is closed (1) or not (0).

$\eta_{ij,t}^{s}$: Binary variable indicates whether the line ij is maintained (1) or not (0).

$\mu_{ij,t}^{s}$: Binary variable indicates whether the state of line ij is closed (1) or not (0).

$w_{ij,t}^{s}$: Assisted binary variable.

$\Delta P_{j,t}^{d}$/$\Delta Q_{j,t}^{d}$: Active/reactive load shedding.

$P_{j,t}^{s,g}$/$Q_{j,t}^{s,g}$: Active/reactive power output of generators.

$P_{ij,t}^{s}$/$Q_{ij,t}^{s}$: Active/reactive power flow.

$P_{j,t}^{s,pv}$: Active power output of photovoltaic generators at time *t*.

$f_{ij,t}^{s}$: Fictitious power flow.

$U_{j,t}^{s}$: Actual voltage magnitude of bus *j*.

$pr_{ij,t}^{s}$: State failure probability of line ij at scenario *s*.
